# *E. adenophorum* induces Cell Cycle Arrest and Apoptosis of Splenocytes through the Mitochondrial Pathway and Caspase Activation in Saanen Goats

**DOI:** 10.1038/srep15967

**Published:** 2015-11-03

**Authors:** Yajun He, Quan Mo, Yanchun Hu, Weihong Chen, Biao Luo, Lei Wu, Yan Qiao, Ruiguang Xu, Yancheng Zhou, Zhicai Zuo, Junliang Deng, Wei He, Yahui Wei

**Affiliations:** 1 Key Laboratory of Animal Disease and Human Health of Sichuan Province, College of Veterinary Medicine, Sichuan Agricultural University, Sichuan Province, Wenjiang 611130, China; 2 Key Laboratory of Resource Biology and Biotechnology in Western China, School of Life Science, Northwest University, Xi’an 710069, China

## Abstract

The precise cytotoxicity of *E. Adenophorum* in relation to the cell cycle and apoptosis of splenocytes in Saanen goats remains unclear. In the present study, 16 Saanen goats were randomly divided into four groups, which were fed on 0%, 40%, 60% and 80% *E. adenophorum* diets. The results of TUNEL, DAPI and AO/EB staining, flow cytometry analysis and DNA fragmentation assays showed that *E. adenophorum* induced typical apoptotic features in splenocytes, suppressed splenocyte viability, and caused cell cycle arrest in a dose-dependent manner. However, westernblot, ELISA, qRT-PCR and caspase activity analyses showed that *E. adenophorum*inhibited Bcl-2 expression, promoted Bax translocation to the mitochondria, triggered the release of Cyt*c* from the mitochondria into the cytosol, and activated caspase-9 and -3 and the subsequent cleavage of PARP. Moreover, in *E. adenophorum*-induced apoptosis, the protein levels of Fas, Bid, FasL and caspase-8 showed no significant changes. *E. adenophorum* treatment induced the collapse of *ΔΨ*m. Moreover, these data suggested that *E. adenophorum* induces splenocyte apoptosis via the activation of the mitochondrial apoptosis pathway in splenocytes. These findings provide new insights into the mechanisms underlying the effects of *E. adenophorum* cytotoxicity on splenocytes.

*Eupatorium adenophorum* (*E. adenophorum*) is an invasive weed and an important destructive exotic species, representing a major threat to the economy and ecology in some regions of the world^1^. This plant is indigenous to Mexico but has been introduced to places such as Hawaii, New Zealand, India, Nepal, and California^2^. *E. adenophorum* has also recently been identified in Chongqing, Yunnan, Sichuan, Tibet, Guangxi and Taiwan. Previous studies have reported that *E. adenophorum* exhibits extensivebiological activity, such as acaricidal activity^3^,^4^,^5^, antitumor activity^6^,^7^ and anti-inflammatory potential^8^. Moreover, previous reports have indicated that *E. adenophorum* has pneumotoxic and hepatotoxic effects on different species of animals. Additionally, it has been reported that regular ingestion of *E. adenophorum* induces chronic pulmonary disease, primarily in Australia, New Zealand, and the Himalayas^2^,^9^, and the methanolic extract of *E. adenophorum* leaf samples induces hepatotoxicity in albino mice^10^. Furthermore, feeding rats a diet mixed with purified extracts from *E. adenophorum* leaf samples causes hepatotoxicity and cholestasis^11^,^12^, and previous studies have shown that the active *Euptox A* isolated from *E. adenophorum* represents an important toxin showing hepatotoxicity^5^,^13^.

As the largest peripheral lymphoid tissue in the body, the spleen is of vital importance in immune function^14^. Previous studies have demonstratedthat ingestion of the methanolic extract of *E. adenophorum* induces obvious atrophy of the spleen, indicating that *E. adenophorum* represents a potential threat to the immune system^15^. Moreover, intravenous administration of *E. adenophorum* affects the proportion of T cells in the spleen and the levels of tumor necrosis factor (TNF)-*α* in mice^16^.

Apoptosis is a type of cell death observed under various physiological and pathological conditions, including during the immune response, cell homeostasis, and inflammation^17^,^18^. Caspase is a key enzyme in the genesis of apoptosis andplays a central role in the execution of apoptosis. Caspase-3, the executioner caspase, is a key factor in the apoptosis of mammalian cells^19^,^20^. Previous studies have revealed that a great number of stimuli can activate caspase-3, including the activation of caspase-8^21^ and caspase-9^22^. Bcl-2, located on the outer membrane of mitochondria, prevents the activation of caspase-3 and inhibits the intrinsic mitochondrial pathway of apoptosis^23^. Bcl-2 prevents the activation of caspase-3 through inhibition of the release of Cyt*c*, a necessary cofactor for caspase-3 activation, from mitochondria^24^,^25^. Bax, identified as the Bcl-2-interacting protein, opposes Bcl-2 and promotes apoptotic cell death^26^.

The above studies have suggested that *E. adenophorum* might act as an apoptotic inducer in the spleen. However, it has not been demonstrated that *E. adenophorum* inhibits the growth and induces apoptosis of splenocytes. In the present study, we investigated the cytotoxic effects of *E. adenophorum* on splenocytesin Saanen goats and detected apoptosis-inducing effects at both the cell andtissue levels, in addition to examining effects on cell cycle progression, to illuminate the potential mechanisms involved in *E. adenophorum*-induced spleen toxicity in goats.

## Results

### Cell cycle of splenocytes

**Figure 1:**
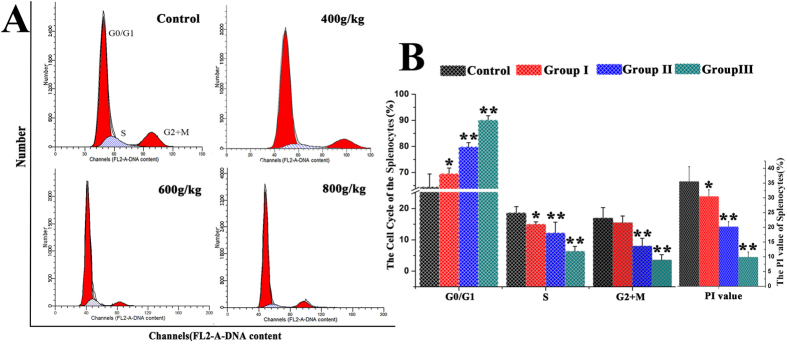
DNA histogram of the splenocyte cell cycle. **(A)**Saanen goats were treated with different doses of *E. adenophorum* for 3 months. Subsequently, the DNA histogram of the splenocyte cell cycle was analyzed through flow cytometry with PI staining. **(B)**The G0/G1%, S% and G2 + M% phases of the splenocytes were analyzed using flow cytometry. The flow cytometric histograms are representative of 3 separate experiments. Proliferating index (PI) value = [S + (G_2_ + M)]/[(G0/G1) + S + (G_2_ + M)] × 100%. The data are presented as the means ± SD of three independent experiments. *p < 0.05 and **p < 0.01, compared with the control group.

The distribution of splenocytes in different phases of the cell cycle was analyzed through flow cytometry (Fig. 1A). After feeding Saanen goats on the *E. adenophorum* diets, the proportion of splenocytes in G_0_/G_1_ phase was substantially increased in the experimental groups. The percentages of splenocytes in S, G_2_ + M and PI phases were significantly decreased in the 600 and 800 g/kg groups and were markedly decreased in group I compared with the control, indicating the occurrence of G0/G1 phase arrest compared with the control group (Fig. 1B). These data suggested that *E.adenophorum* inhibited splenocyte growth in Saanen goats through inhibition of the G0/G1 to S phase transition in the cell cycle.

### Detection of apoptotic splenocytes

**Figure 2:**
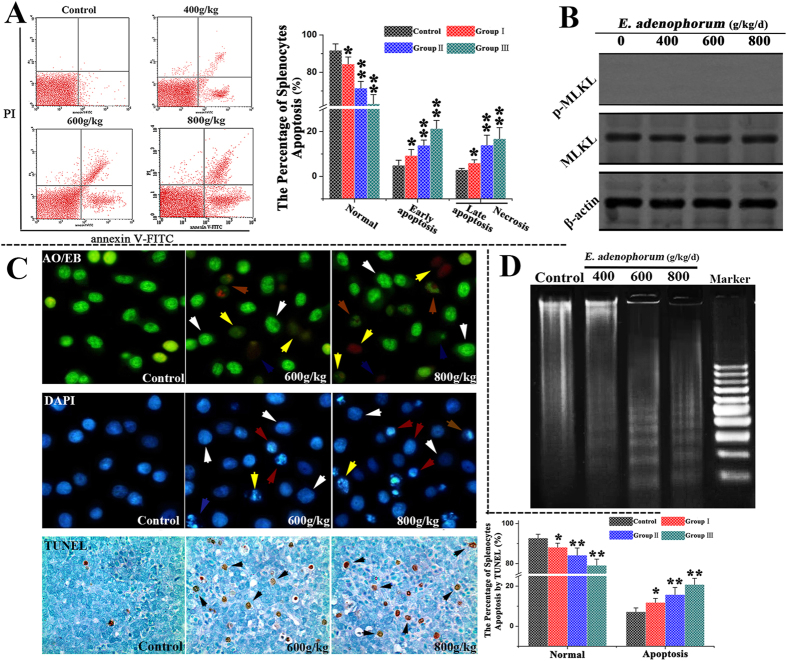
*E. adenophorum* administration induces apoptosis in splenocytes. **(A)** Scattergram of apoptotic splenocytes. The splenocytes were analyzed for apoptosis through flow cytometry based on Annexin V and PI staining.The percentage of splenocyte apoptosis(%). *E. adenophorum* significantly induced apoptosis in splenocytes. **(B)** MLKL and p-MLKL protein levels were measured to detect programmed necrotic cell death through western blot analysis. **(C)** Detection of apoptotic splenocytes through DAPI and AO/EB staining and TUNEL assays. Representative spleen sections from Saanen goats were analyzed in TUNEL assays to detect apoptotic cell death. The number of TUNEL-positive cells (black indicated arrows) in the spleen was counted from five random microscopic fields. Magnification, 400×. Nuclear morphological changes in splenocytes were observed using a fluorescence microscope after DAPI (200×) and AO/EB staining (400×). Normal cells(white arrow), early apoptosis(red arrow), late apoptosis(yellow arrow), and necrosis (blue arrow). **(D)** Induction of DNA fragmentation. DNA isolated from *E. adenophorum*-treated splenocytes was subjected to 2% agarose gel electrophoresis, followed by the visualization of bands and photography. The data are presented as the means ± SD of three independent experiments. *p < 0.05 and **p < 0.01 compared with the control group.

The apoptosis of splenocytes was further examined. The rate of apoptosis in the splenocytes was assessed through TUNEL stainingand flow cytometry using Annexin V/PI staining. The results indicated that the percentage of normal splenocytes was markedly decreased compared withthe control. The percentages of apoptotic splenocytes in groups II and II were significantly increased with an increasing percentage of *E. adenophorum*. Moreover, the proportion of apoptotic splenocytes was positively correlated with the dose of *E. adenophorum* (Fig. 2A). The mixed lineage kinase domain-like protein (MLKL) has emerged as the key cellular component in programmed necrotic cell death. To determine whether programmed necrotic cell death was associated with *E. adenophorum* administration, MLKL phosphorylation was analyzed through western blotting. The results indicated that *E. adenophorum* failed to increase the protein levels of p-MLKL (Fig. 2B). The TUNEL assay was used to detect DNA strand breaks occurring prior to nuclear fragmentation^27^, and TUNEL-positive cells were quantified through manual counting. In the present study, significant apoptosis was observed through the TUNEL assay. However, significant numbers of TUNEL-positive cells were not observed in the control group. The quantification results indicated the occurrence of 15.78% and 20.82% TUNEL-positive cells at doses of 600 and 800 g/d *E. adenophorum*, respectively. DAPI and AO/EB staining, which were used to examine nuclear morphology, showed that the splenocytes of the control group did not presentsignificant changes in cell nuclei and cell membrane integrity (white arrow, Fig. 2C). However, typical apoptotic nuclei were observed in the splenocytes of the experimental groups, including the occurrence of chromatin condensation (red arrow, Fig. 2C), nuclear fragmentation and cell membrane destruction (yellow arrow, Fig. 2C). Moreover, the DNA fragmentation assay revealed characteristic ladder patterns in splenocytes, and the DNA ladder was more evident in *E. adenophorum*–treated splenocytes (Fig. 2D). These results demonstrated that *E. adenophorum* induced splenocyte apoptosis in a dose-dependent manner but did not induce programmed necrotic cell death.

### Activation of caspases-9 and -3, but not caspase-8, is involved in apoptosis

**Figure 3:**
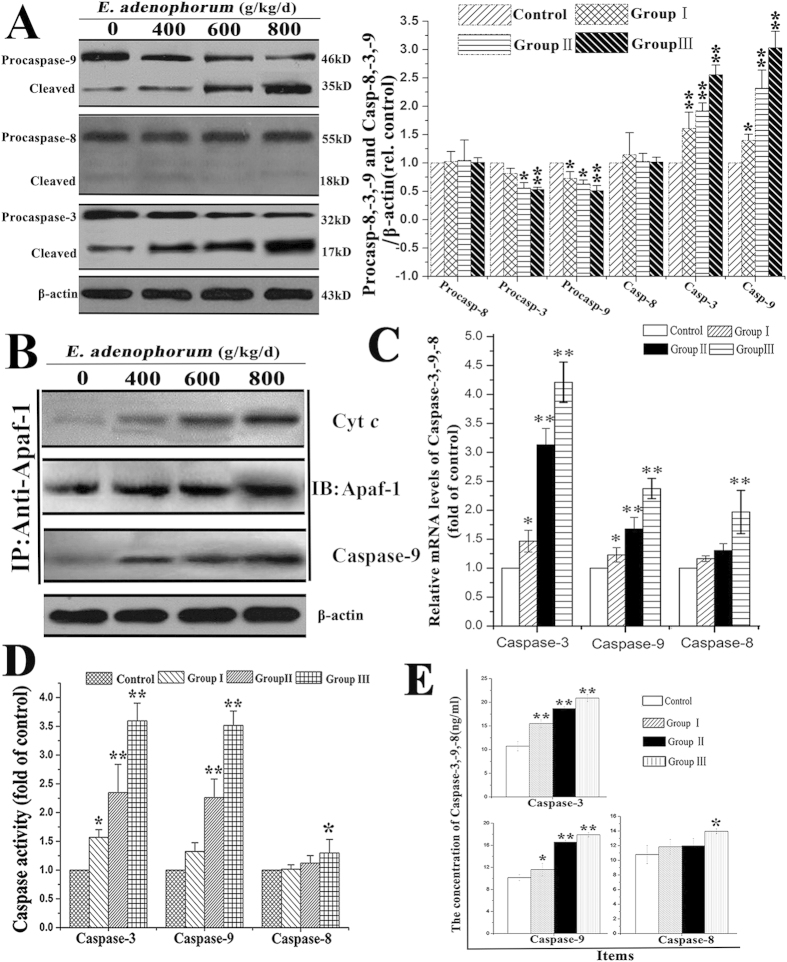
*E. adenophorum*-induced apoptosis is mediated through the activation of caspase-9 and caspase-3. **(A)**The protein levels of procaspases-3, -8 and -9 and the cleaved forms of these proteins. The expression of apoptosis-related proteins, including caspases-3, -9, and -8, is shown using β-actin as a control, as detected through western blot analysis. Densitometric quantification of the ratio of procaspases-3, -8 and -9 and the cleaved forms of them to β-actin. Data are normalized to control. **(B)** *E. adenophorum* administration induced apoptosome formation. Protein extracts from splenocytes were collected and used in immunoprecipitation assays against Apaf-1. The levels of caspase-9 and Cyt*c* were detected through western blotting to indicate the formation of the apoptosome complex. **(C)** Relative mRNA levels of caspases-3, -8 and -9. The Saanen goats were treated with different doses of *E. adenophorum* for 3 months, and mRNA was then extracted from splenocytes and used for qRT-PCR analysis. *E. adenophorum*induced the activation of caspases-3 and -9. **(D)** Caspase activity in *E. adenophorum*-treated splenocytes. The BCA assay was used to verify equalamounts of protein, and the enzymatic activities of caspases-8, -9, and -3 were measured using the colorimetric assay kit.**(E)** The concentration of caspases-3, -8, and -9 in splenocytes was measured through ELISA. The data are presented as the means ± SD of three independent experiments. *p < 0.05 and **p < 0.01, compared with the control group.

Caspases are the central components in the execution of apoptosis. Caspase-8 and -9 are initiator caspases in the death receptor and mitochondrial pathways, respectively. Caspase-3, the downstream caspase of caspases-8 and -9, is the key executioner caspase in the apoptosis pathway. The contributions of caspases-8, -9 and -3 to *E. adenophorum*-induced apoptosis were measured through qRT-PCR, caspase activity assays, ELISA and western blot assays. Western blot analysis showed that the levels of full-length procaspase-9 and procaspase-3 were decreased, and their cleaved forms were increased with increasing levels of *E. adenophorum*. However, the full-length and cleaved forms of procaspase-8 did not show any differences (Fig. 3A). Released Cyt*c* typically combines with Apaf-1 and procaspase-9 to form the apoptosome in the presence of ATP in the cytoplasm, resulting in the activation of caspase-9. To determine whether *E. adenophorum* promoted the formation of the apoptosome, cell lysates were immunoprecipitated with an anti-Apaf-1 antibody and subsequently subjected to western blot analysis with anti-caspase-9 and anti-Cyt*c*antibodies. The results showed that Apaf-1 interacted with caspase-9 and Cyt*c* (Fig. 3B). Moreover, qRT-PCR revealed that the activation of caspases-3 and -9 was evidently inducedby*E. adenophorum*, whereas the activation of caspase-8 was not induced in groups I and II (Fig. 3C). The caspase molecules involved in *E. adenophorum*-induced apoptosis were analyzed to measure the activities of caspases-8, -9, and -3 using colorimetric assay kits. Here, we showed that *E. adenophorum* significantly induced the activation of caspases-9 and -3 but failed to induce the activation of caspase-8, except at a dose of 800 g/kg/d (Fig. 3D). Compared with the control, the concentrations of caspase-3 and -9, but not that of caspase-8, showed a significant increase (Fig. 3E). These results suggested that *E. adenophorum*-induced apoptosis primarily depends on the activation of caspase-9 and caspase-3, but not caspase-8.

### The mitochondrial apoptotic pathway is activated in *E. adenophorum*-induced apoptosis

**Figure 4:**
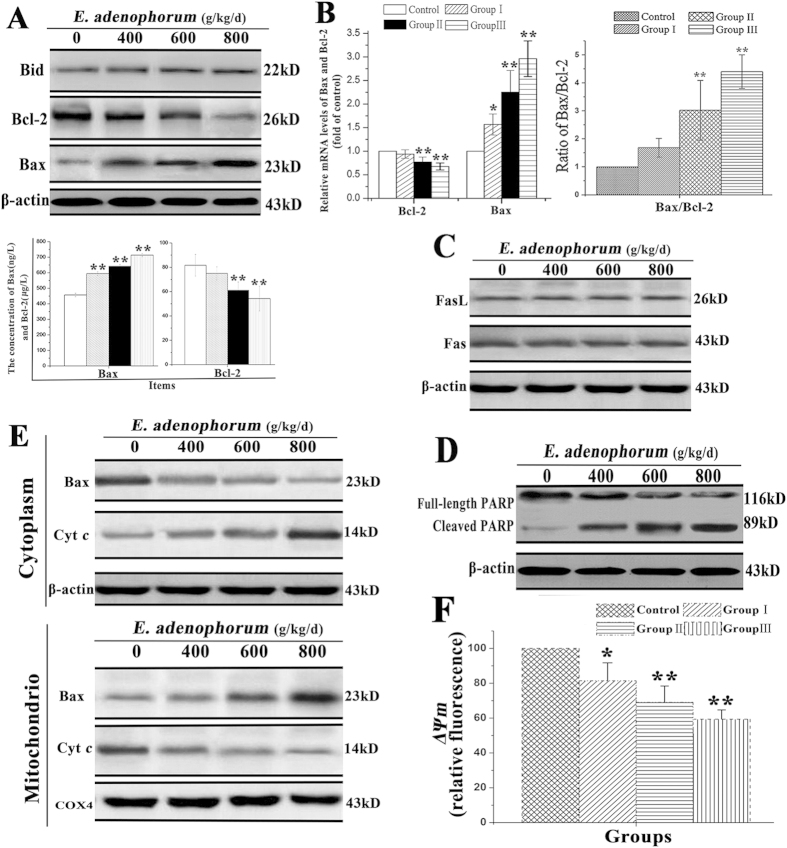
Thesplenocyte apoptosis induced through *E. adenophorum* was mediated through the mitochondrial pathway. **(A)** *E. adenophorum* treatment did not promote the cleavage of Bid but did increase the protein levels of Cyt*c* and Bax and decreased the protein level of Bcl-2. The concentrations of Bax and Bcl-2 in splenocytesweremeasured through ELISA. The data are presented as the means ± SD of three independent experiments. *p < 0.05 and **p < 0.01 compared with the control group. **(B)** *E. adenophorum* treatment decreased the relative mRNA level of Bcl-2 but increased the level of Bax, resulting in an increased ratio of Bax/Bcl-2. **(C)** The protein levels of Fas or FasL were measured through western blotting, and the results did not reveal any changes. **(D)**The splenocytes were subjected to western blot analysis to detect total and activated PARP. **(E)** *E. adenophorum* induced Bax translocation and Cyt*c* release. The proteins in the cytosolic and mitochondrial fraction were collected and subsequently detected through western blotting. COX IV and β-actin were used as internal controls for the mitochondrial and cytosolic fractions, respectively. **(F)** *E. adenophorum* treatment induced collapse of *ΔΨm*. The cell suspension was filtered through 300-mesh nylon and then stained with JC-1, followed by FCM analysis.

The two distinct signaling pathways, the death receptor pathway and the mitochondrial pathway, are involved in apoptosis. The death receptor pathway is typically triggered through the ligation of death receptors, such as Fas or Fas ligand (FasL), which recruits fas-associated proteins with a death domain and procaspase-8 to form a death-inducing signaling complex, leading to the proteolytic activation of caspase-8. Studies have known that activated caspase-8 cleaves Bid to form truncated Bid, which activates the mitochondria-mediated apoptotic pathway^28^. Mitochondria play a vital role in apoptosis induced by chemical agents^29^,^30^. The pro-apoptotic and anti-apoptotic members of the Bcl-2 family, such as Bcl-2 and Bax, regulate mitochondrial membrane integrity. Bcl-2 protects cells from the induction of apoptosis through interacting with Bax, blocking the release of Cyt*c* from the mitochondria to the cytosol. The expression levels of Cyt*c*,Fas, FasL, Bcl-2, Bid and Bax were examined to explore the effects of *E. adenophorum* on splenocytes through western blot, qRT-PCR and ELISA analyses. The results showed that *E. adenophorum*increased the protein levels of Bax and Cyt*c* and decreased the protein level of Bcl-2 in a dose-dependent manner in splenocytes. However, the level of Bid did not show significant variations, consistent with the results for caspase-8, suggesting that the activation of the mitochondrial pathway was independent of the activation of caspase-8 and Bid (Fig. 4A). qRT-PCR indicated that the relative mRNA level of Bax increased, while that of Bcl-2 decreased, resulting in a change in the Bax/Bcl-2 ratio, which subsequently activated the mitochondrial pathway (Fig. 4B). Consistent with the results for caspase-8, the protein levels of Fas and FasL were not affected by *E. adenophorum*, as detected through western blotting, suggesting that the Fas-mediated death receptor pathway was not activated in thesplenocytesby *E. adenophorum* (Fig. 4C). PARP is an indicator of caspase-3 activation during apoptosis. Western blotting showed that PARP was cleaved from a 116 kDa fragment to an 85 kDa fragment during *E. adenophorum*-induced apoptosis (Fig. 4D). The protein extracts of both the mitochondrial and cytosolic fractions were used to determine the location of Bax and Cyt*c* in splenocytes. A dose-dependent decrease in mitochondrial Cyt*c* and a concomitant increase in the cytosolic fraction were also observed. Accordingly, translocation of Bax from the cytosol to mitochondria was observed (Fig. 4E). *ΔΨm* is determined by the balance of pro-apoptotic and anti-apoptotic Bcl-2 family members, such as Bax and Bcl-2. To determine whether *E. adenophorum* treatment activates mitochondriamediated apoptotic pathway, we used JC-1 as fluorescence probe to evaluate changes in *ΔΨm* by flow cytometry. The result showed that *E. adenophorum* treatment significantly induced the collapse of *ΔΨm* (Fig. 4F). These results suggested that *E. adenophorum*-induced apoptosis primarily occurred through activation of the mitochondrial pathway.

## Discussion

*E. adenophorum* is a noxious invasive weed that is found worldwide^1^. According to previous studies, the ingestion of freeze-dried *E. adenophorum* leaf powder as a diet supplement induces hepatotoxicity in mice as well as anorexia, rumination suspension and photosensitization in cattle^31^ and chronic pulmonary disease in horses^2^. These observations provide a rationale for exploring *E. adenophorum* as a cause of splenic toxicity and an inducer of apoptosis in the splenocytes of Saanen goats.

Flow cytometry is frequently used to monitor early apoptosis^32^, and the flow cytometry results of the present study showed that the *E. adenophorum* increased the numbers of apoptotic splenocytes, indicating that *E. adenophorum* reduced the survival and inhibited the growth of the splenocytes through the induction of apoptosis and cell cycle arrest, suggesting that the splenocytes of Saanen goats are sensitive to *E. adenophorum*. Our results also showed that *E. adenophorum* induced splenocyte apoptosis with typical morphological characteristics, including cellular shrinkage, chromatin condensation, and DNA fragmentation. The mitochondria-mediated apoptotic pathway requires the translocation of Cyt*c* from the mitochondria to the cytosol and the formation of a large multiprotein complex comprising Cyt*c*, Apaf-1 and procaspase-9. In the present study, we observed that *E. adenophorum*-induced apoptosis involved the activation of caspase-9 and -3. However, *E. adenophorum* did not activate Fas and FasL and the downstream protein capase-8. Thus, these results demonstrate that apoptosis is dependent on the activation of caspases-9 and -3, and *E. adenophorum* failed to activate the death receptor-mediated caspase-8 pathway in the present study. In splenocytes, Cyt*c* released from dysfunctional mitochondria into the cytosol forms apoptosomes together with Apaf-1 and procaspase-9, followed by the activation of caspases-9 and -3 and the cleavage of PARP. In the present study, *E. adenophorum* failed to activate Fas, FasL and Bid, suggesting that the mitochondria-mediated apoptosis pathway is activated through *E. adenophorum* administration and that *E. adenophorum* induces apoptosis through Cyt*c*-mediated and caspase-dependent pathways. Both the protein and mRNA levels of *E. adenophorum* decreased the levels of Bcl-2, while those of Bax increased. Additionally, *E. adenophorum* promoted the translocation of Bax from the cytoplasm to the mitochondria and Cyt*c* from the mitochondria to the cytoplasm, indicating that the mitochondrial pathway was activated. The down-regulation of Bcl-2 and up-regulation of Bax demonstrated the dysregulation of the associated molecules Bcl-2 and Bax and the activation of caspases-3 and -9^33^,^34^. The dysregulation of the mitochondria integrity-associated molecules Bcl-2 and Bax suggested that activation of the mitochondrial pathway is the main event during apoptosis. Furthermore, In response to apoptotic stimuli, Bax translocates to the mitochondria and inserts into the outer mitochondrial membrane, resulting in the collapse of *ΔΨm*^35^. Here we showed that *E. adenophorum* induced the collapse of *ΔΨm*, so the translocation of Bax may be associated to the collapse of *ΔΨm* and release of mitochondrial pro-apoptotic proteins. The degree of apoptosis mediated through the mitochondria could reflect cell cycle regulation, which is a key regulatory mechanism underlying cell growth^36^. The cell cycle analysis and PI values showed that *E. adenophorum* intake could effectively inhibit the growth of splenocytes through cell cycle arrest, similar to injury of the spleen, as previously reported^37^.

The results of the present study demonstrated that *E. adenophorum* significantly inhibits the growth of splenocytes through G0/G1-phase cell cycle arrest and the induction of apoptosis. *E. adenophorum* down-regulated Bcl-2, promoted Bax translocation into the mitochondria, and activated the mitochondria-dependent apoptotic pathway, resulting in Cyt *c* release into the cytosol, followed by caspase-9 and caspase-3 activation and PARP cleavage. Moreover, *E. adenophorum* induced apoptosis and spleen impairment through the induction of mitochondrial dysfunction in splenocytes. Additionally, mitochondrial dysfunction was considered to be responsible for the apoptosis observed in splenocytes. This study provides new insights into the mechanisms underlying splenocyte apoptosis through *E. adenophorum* infection in Saanen goats.

## Materials and Methods

### Ethics statement

All experimental procedures and animal care performed in the present study were approved according to the recommendations of the Guide of the Sichuan Agricultural University Animal Care and Use Committee(Sichuan Agricultural University, Sichuan, China) under permit NO. DKY-B20100805, and all efforts were made to minimize suffering. The field studies did not involve endangered or protected species. The Saanen goats were housed at the experimental farm of the Animal Nutrition Institute of Sichuan Agricultural University.

### Experimental animals and plant materials

A total of 16 Saanen goats (12 males and 4 females, with an average weight and age of 25.34 ± 1.11 kg and 3.15 ± 0.13 months, respectively) were randomly selected for the experiments and divided into four groups. The control group and groups I, II, and III were fed 0%, 40% (i.e., 400 g/kg), 60% (i.e., 600 g/kg), and 80% (i.e., 800 g/kg) *E. adenophorum* diets, respectively, twice a day for 3 months, according to Sahoo^38^. The Saanen goats were fed 1 kg/d of the feedstuffs. Ryegrass and water were freely available. *E. adenophorum*leaves were collected from cropland in Xichang, Sichuan Province. Subsequently, the collected plant leaves were washed, dried and ground at room temperature to generate dry powder.

### Cell cycle detection through flow cytometry

Thespleens were immediately removed and minced using scissors to form a cell suspension that was filtered through a 300-mesh nylon screen. The cells were washed twice with cold PBS (pH 7.2–7.4) and subsequently suspended in PBS (Cat. No. 51-66121E, BD) at a concentration of 1 × 10^6^ cells/ml.A total of 500 μl of the solution was transferred to a 5 ml culture tube, followed by centrifugation (200 × g). After the cell suspension was permeabilized with 1 ml of 0.25% Tritonx-100 for 20 min at 4 °C, the cells were washed with phosphate buffered saline, and 5 μl of propidium iodide (Cat. No. 51-66211E, BD) was subsequently added. The cells were then gently vortexed and incubated for 30 min at 4 °C in the dark. Finally, 500 μl of PBS was added to each tube, and the cell cycle phases were analyzed through flow cytometry (BD FACSCalibur, San Jose, CA, USA) within 45 min.

### Annexin-V/PI apoptosis detection

The spleens were immediately collected from each of the Saanen goats. The cell suspension was filtered through 300-mesh nylon and washed twice with cold PBS, and the cells were subsequently suspended in 1× binding buffer (Cat. No. 51-66121E) at a concentration of 1 × 10^6^ cells/mL. A total of 100 μL of the solution was transferred to a 5 mL culture tube, and 5 μL of Annexin V-FITC (Cat. No. 51-65874X) and 5 μL of PI (Cat. No. 51-66211E) were added. The cells were gently vortexed and incubated for 15 min at RT (25 °C) in the dark. Subsequently, 400 μL of 1× binding buffer was added to each tube and analyzed through flow cytometry (BD FACSCalibur) within 1 h.

### qRT-PCR analysis of the mRNA expression of Bax, Bcl-2, and caspases-3, 8, and 9

Total RNA was isolated from spleen powder (50 mg) using TRIzol (Aidlab, China) according to the manufacturer’s instructions. The synthesis of single-stranded cDNA from 5 μg of RNA was performed according to the TUREscript 1st-strand cDNA Synthesis Kit(Aidlab, China), and the mRNA was reverse transcribed into cDNA. The cDNA was used as a template for qRT-PCR analysis. Relative gene expression was defined as the ratio of target gene expression to β-actin gene expression^39^. The gene expression values in the control group were used for the calibration of gene expression. The results were analyzed using the 2^−△△Ct^ method^40^. The primer sequences are shown in Table 1.

### DNA fragmentation assay

Both control and *E. adenophorum-*treated splenocytes were collected and washed with PBS. DNA extraction was performed according to previous studies^41^. After dissolving the DNA in TE buffer, it was subjected to 2% agarose gel electrophoresis for DNA fragmentation analysis.

### Western blot analysis

The splenocyteswere harvested and washed with ice-cold PBS, followed by lysis in ice-cold RIPA lysis buffer (Beyotime Inst. Biotech, Beijing, China) containing 1 mmol/L PMSF. The protein concentrations were calculated using BCA assay kits (Pierce). A total of 20 μg of the total cellular protein was subjected to 12% SDS-PAGE and transferred to a PVDF membrane (Millipore, Atlanta, GA, USA). The membrane was blocked with 5% defatted milk powder at room temperature for 1 h followed by immunoblotting with primary antibodies at 4 °C overnight and then incubation with HRP-conjugated secondary antibodies at room temperature for 1 h. Following each step, the membranes were washed five times with PBS-T for 3 min. Finally, the blots were developed using the enhanced chemiluminescence (ECL) system (Pierce).

### Analysis of Bax, Bcl-2, and caspase-3, 8, and 9 concentrations through ELISA

Four Saanen goats from each group were euthanized after a formal trial of 3 months. Spleen samples: After harvesting the spleens, the samples were weighed, washed with PBS (pH 7.2-7.4), rapidly frozen with liquid nitrogen, and maintained at 2–8 °C after melting, followed by the addition of PBS (pH 7.4). The samples were then homogenized using grinders and centrifuged for 20 min at 2500 rpm, and the supernatant was removed. The concentrations of Bax, Bcl-2, and caspases-3, 8, and 9 in the spleen were assayed using a double-antibody sandwich enzyme-linked immunosorbent assay (ELISA) (R&D Systems, China).

### Assessment of the mitochondrial transmembrane potential (*ΔΨm*)

The transmembrane potential, *ΔΨm*, was analyzed using the JC-1 Mitochondrial Potential Detection Kit (Biotium Inc., Hayward, CA, USA). The cell suspension was filtered through 300-mesh nylon, washed twice with cold PBS and stained with 5,5′,6,6′-tetrachloro-1,1′,3,3′ tetraethylbenzimidazolcarbocyanine iodide (JC-1; Molecular Probes) in PBS for 15 min at room temperature in the dark, followed by flow cytometric analysis.

### Caspase activity measurement

Caspase activity was measured using colorimetric assay kits (BioVision, Inc., Mountain View, California, USA), according to the manufacturer’s instructions. Briefly, the splenocytes were harvested and incubated in ice-cold cell lysis buffer for 30 min on ice. The supernatants were collected, and the protein concentrations were determined using the BCA Protein Assay Reagent (Pierce, Rockford, IL, USA). Equivalent amounts of proteins for each sample were incubated with the appropriate caspase substrate. After incubation at 37 °C for 4 h, protease activity was determined at 405 nm using a microplate spectrophotometer (Bio-Tek Instruments, Inc., Winooski, USA).

### TdT-mediated dUTP nick-end labeling (TUNEL) assay

The spleen tissues were fixed in 4% paraformaldehyde, embedded in paraffin and cut into 6 μm sections. A TUNEL assay was then conducted to examine DNA fragmentation using an *in situ* cell death detection kit (Vazyme, Piscataway, NJ, USA) according to the manufacturer’s instructions. After mounting the TUNEL-positive cells, the nuclei were counterstained with DAPI, and the sections were observed at ×1000 magnification under a Nikon microscope (Nikon Inc., Japan).

### Apoptosis assessment through DAPI and AO/EB staining

The spleens were harvested and minced using scissors to form a cell suspension that was filtered through a 300-mesh nylon screen. For DAPI staining, the splenocytes were fixed with 80% ethanol at room temperature for 30 min. The fixative was then removed, and the splenocytes were washed 3 times with PBS and subsequently incubated with DAPI (1 μg/ml) for 45 min at room temperature in the dark. For AO/EB staining, unfixed cells were loaded using 100 μl of freshly prepared AO/EB staining solution (100 μg/ml) and immediately observed under a Nikon fluorescence microscope (Nikon Inc., Japan) within less than 20 min.

### Statistical analysis

All data are expressed as the means ± SD of three independent experiments. Statistical analyses were performed to compare the experimental groups with the control group through one-way analysis of variance (ANOVA), followed by the Tukey-Kramer multiple comparison test with an equal sample size. All statistical analyses were performed using a commercially available statistical software package (SPSS15.0, SPSS Inc., USA).

## Additional Information

**How to cite this article**: He, Y. *et al.* *E. adenophorum* induces Cell Cycle Arrest and Apoptosis of Splenocytes through the Mitochondrial Pathway and Caspase Activation in Saanen Goats. *Sci. Rep.* **5**, 15967; doi: 10.1038/srep15967 (2015).

## Figures and Tables

**Table 1:** The primers used for qRT- PCR.

Items	Sense (5′ - to- 3′’)	Antisense (5′ - to- 3′)
Bax	CCTGCTTCTTTCTTCATCGG	AGGTGCCTGGACTCTTGGGT
Bcl-2	GGCTGGGATGCTTTGTG	GAGCAGTGCCTTCAGAGACAGC
Caspase-3	GCAGCAAACCTCAGGGAAAC	GGTTTCCCTGAGGTTTGCTG
Caspase-8	AAGAACGAGCCTCAGTAATC	GGATTACTGAGGCTCGTTCT
Caspase-9	GAAGACCAGCAGACAAGC	TGAATCCTCCAGAACCAA
β-actin	CCTGCTTCTTTCTTCATCGG	AGGTGCCTGGACTCTTGGGT
